# The density of parasympathetic axons is reduced in the exocrine pancreas of individuals recently diagnosed with type 1 diabetes

**DOI:** 10.1371/journal.pone.0179911

**Published:** 2017-06-19

**Authors:** Marcus Lundberg, Andreas Lindqvist, Nils Wierup, Lars Krogvold, Knut Dahl-Jørgensen, Oskar Skog

**Affiliations:** 1 Department of Immunology, Genetics and Pathology, Uppsala University, Uppsala, Sweden; 2 Lund University Diabetes Centre, Lund University, Malmö, Sweden; 3 Division of Paediatric and Adolescent Medicine, Oslo University Hospital, Oslo, Norway; 4 Faculty of Medicine, University of Oslo, Oslo, Norway; Children's Hospital Boston, UNITED STATES

## Abstract

To elucidate the etiology of type 1 diabetes, the affected pancreas needs to be thoroughly characterized. Pancreatic innervation has been suggested to be involved in the pathology of the disease and a reduction of sympathetic innervation of the islets was recently reported. In the present study, we hypothesized that parasympathetic innervation would be altered in the type 1 diabetes pancreas. Human pancreatic specimens were obtained from a unique cohort of individuals with recent onset or long standing type 1 diabetes. Density of parasympathetic axons was assessed by immunofluorescence and morphometry. Our main finding was a reduced density of parasympathetic axons in the exocrine, but not endocrine compartment of the pancreas in individuals with recent onset type 1 diabetes. The reduced density of parasympathetic axons in the exocrine compartment could have functional implications, e.g. be related to the exocrine insufficiency reported in type 1 diabetes patients. Further studies are needed to understand whether reduced parasympathetic innervation is a cause or consequence of type 1 diabetes.

## Introduction

Despite intense research, the etiology of type 1 diabetes remains undetermined. Most of the current knowledge is derived from animal models, due to scarce access to human biopsies, and several aspects of the type 1 diabetes pancreas remain uninvestigated. To unravel the etiopathogenesis of type 1 diabetes, we need to establish features that distinguish the type 1 diabetes pancreas [1, 2].

The autonomic (parasympathetic and sympathetic) nervous system has an evident function in regulating islet hormone secretion in man. Administration of drugs that inhibit the neurotransmission across autonomic ganglia impairs the insulin response during the cephalic phase [3] and pancreas denervation affects insulin secretion [4]. Furthermore, vagotomy, autotransplantation and transplantation of pancreas, all reduce pancreatic polypeptide (PP) secretion in response to hypoglycemia [5–7]. However, the mechanisms by which the autonomic nervous system regulates islet function remains undetermined.

Already in the 19^th^ century, Paul Langerhans described innervation of the pancreas in rabbits and cats [8]. However, studies on human pancreata were sparse until 2011, when Rodriguez-Diaz et al. described islets to be mainly innervated by sympathetic axons that target endocrine cells [9]. Mundinger et al. recently described the number of sympathetic axons to be reduced in the islets of individuals with recent onset type 1 diabetes [10] and sensory nerve fibers have earlier been described to be important in the etiopathology of diabetes in NOD mice [11].

Previous studies have shown that vasoactive intestinal peptide (VIP)-containing parasympathetic axons can be found within and close to pancreatic islets as well as in the exocrine and other pancreatic tissues [12–15]. However, parasympathetic axons have not been investigated previously in human type 1 diabetes pancreata.

In the present study, we hypothesized that parasympathetic innervation would be altered in the type 1 diabetes pancreas and aimed to examine the density and distribution of parasympathetic axons within the pancreata of type 1 diabetic individuals and matched non-diabetic individuals. The density of parasympathetic axons was assessed in human pancreatic specimens obtained from previously healthy organ donors, organ donors and live patients with recent onset type 1 diabetes included in the DiViD-study [16, 17], and donors with a long history of type 1 diabetes.

## Methods

### Ethics

Collection of pancreatic tissue in the Diabetes Virus Detection study (DiViD) was approved by The Norwegian Governments Regional Ethics Committee. Written informed consent was obtained from all individuals after oral and written information from the diabetologist and the surgeon separately. Consent for organ donation (for clinical transplantation and for use in research) was obtained verbally from the deceased’s next of kin by the attending physician and documented in the medical records of the deceased in accordance with Swedish law and as approved by the Regional Ethics Committee (Dnr 2015/444). None of the donors or patients were from a vulnerable population.

### Human pancreatic specimens

A total of 22 human pancreata, collected between June 2007 and February 2014, were included in the study (Table 1). The first group, referred to as non-diabetic controls, consisted of pancreata collected from seven non-diabetic, brain-dead multiorgan donors. The second group, referred to as recent onset type 1 diabetic individuals, contained eight pancreatic samples. Two of these were from multiorgan donors described previously [17], who died as a consequence of acute onset type 1 diabetes, and six were from patients recruited to the DiViD study. In the DiViD study, pancreatic biopsies consisting of an approximately 3 cm long single piece were resected three to nine weeks after diagnosis of type 1 diabetes (median five weeks) as described previously [16]. The third group consisted of pancreata collected from seven multiorgan donors with a long history of type 1 diabetes. All donors with recent onset type 1 diabetes, and none of the donors with long-standing type 1 diabetes, have previously been diagnosed with insulitis [18, 19] as defined by ≥3 islets with ≥15 CD3+ cells. All pancreatic tissue included in the study was from the tail region of the pancreas and prepared as described previously [16]. The DiViD samples were fixated within 240 seconds after the pancreatic tail resection. All pancreata from brain-dead organ donors were preserved as for transplantation and transported to the islet isolation facility at Uppsala University Hospital in cold preservation solution (UW or HTK). The cold ischemia time from donor perfusion to procurement of biopsies ranged from 04:41 to 18:56 (mean 10:19) hours. Formalin-fixed and paraffin-embedded tissue was used. As the classical markers for parasympathetic axons, such as vesicular acetylcholine transporter (vAChT), appears to be expressed also in islet cells [9] we stained for VIP, which is expressed exclusively in parasympathetic axons [20]. One section (6 μm) from two different paraffin blocks from each of the DiViD pancreata, and one section from each of the donor pancreata, were stained for VIP.

**Table 1:** Clinical characteristics of donors and patients.

Case	Age (years)	Sex	BMI (kg/m^2^)	HbA_1c_ at biopsy % (mmol/mol)
ND-1	37	F	29.4	5.1 (32)
ND-2	20	M	27.8	5.8 (40)
ND-3	21	M	20.1	5.2 (33)
ND-4	25	M	22.9	5.9 (41)
ND-5	27	M	26.0	5.7 (39)
ND-6	24	F	25.3	5.9 (41)
ND-7	35	F	24.7	-
DIVID1	25	F	21.0	6.7 (50)
DIVID2	24	M	20.9	10.3 (89)
DIVID3	34	F	23.7	7.1 (54)
DIVID4	31	M	25.6	7.4 (57)
DIVID5	24	F	28.6	7.4 (57)
DIVID6	35	M	26.7	7.1 (54)
Acute onset 1	40	M	27.2	-
Acute onset 2	29	M	24.2	10.4 (90)
LS-1	17	M	20.0	6.1 (43)
LS-2	36	F	20.9	7.4 (57)
LS-3	59	F	23.9	8.2 (66)
LS-4	68	M	30.2	8.0 (64)
LS-5	24	M	27.5	8.3 (67)
LS-6	47	F	27.6	7.4 (57)
LS-7	25	M	22.8	8.9 (74)

ND = Non-diabetic control. *DIVID* and *Acute onset* are recent onset type 1 diabetic individuals. LS = Long-standing type 1 diabetic individual.

### Immunofluorescence

Antibodies were diluted in PBS (pH 7.2) containing 0.25% BSA and 0.25% Triton X-100. Sections were incubated with primary antibody (VIP: M7852, 1:3000, EuroDiagnostica, Malmö, Sweden) overnight at 4°C, followed by rinsing in PBS with Triton X-100 for 2 x 10 min. Thereafter, a donkey anti-rabbit Cy2-conjugated secondary antibody (1:400; Jackson, West Grove, PA) was applied to the sections. Incubation period was 1 h at room temperature. Sections were again rinsed and then mounted on slides using PBS-glycerol (1:1).

### Microscope analysis

**Fig 1:**
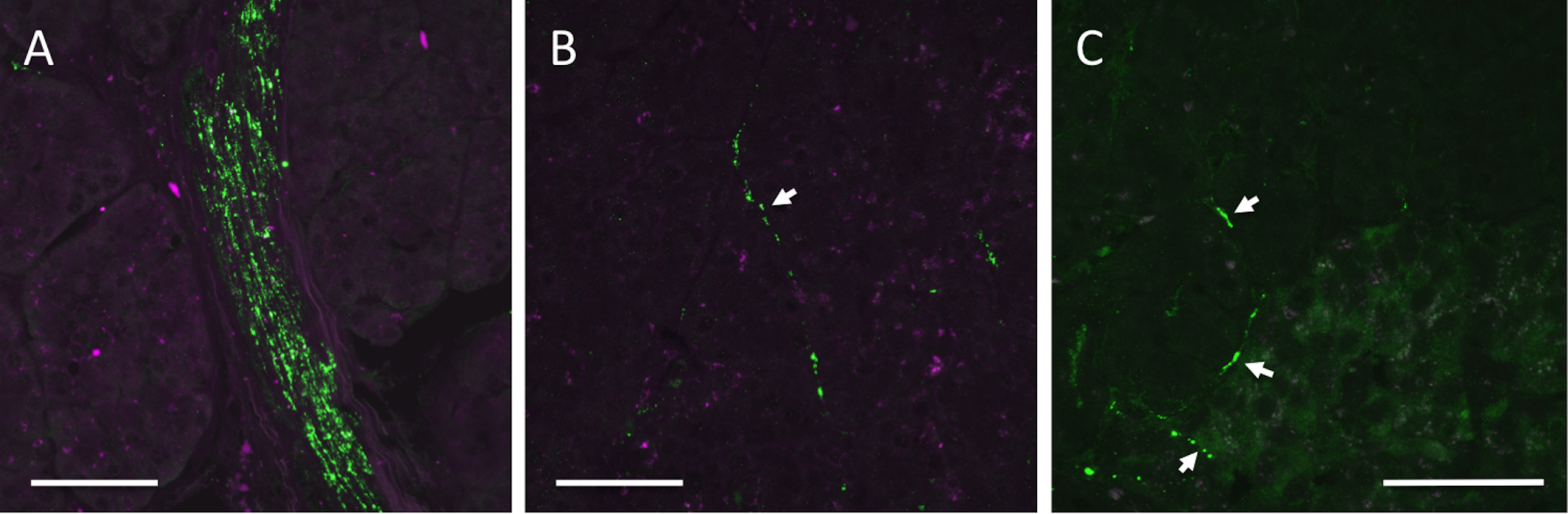
Representative images of VIP-stained parasympathetic axons. Parasympathetic axons were dense in nerve bundles (A). A parasympathetic axon in exocrine tissue (B). Three parasympathetic axons close to, at the periphery, and inside an islet (C). The right bottom corner in (C) is an islet. White arrows point toward axons. The scale bars correspond to 50 μm.

All analyzes were performed by the same observer unaware of the origin of the slides. Axons were counted manually using a Nikon Eclipse Ti microscope (Nikon, Tokyo, Japan) and an Olympus BX60 microscope (Olympus corporation, Tokyo, Japan) at 400X magnification. Representative pictures were taken with a confocal microscope LSM700 microscope (Zeiss, Germany) and illustrated as maximum intensity projected images. VIP is enriched in vesicles. To exclude possible artifacts in the tissue, VIP immunoreactive axons were defined by a minimum of three closely aligned immunoreactive spots (see Fig 1 for representative pictures). VIP immunoreactive axons were searched for in the entire section, and the tissue type where the axons were found was noted. Axons located in nerve trunks were excluded from the analysis to avoid skewing the results due to random appearance of an axon-rich nerve trunk in a section. Islets were identified based on autofluorescence and morphology (see Fig 1C and S1C Fig for representative pictures). Axons were investigated in relation to islets in three ways; 1) within islets and surrounded by endocrine cells, 2) at the periphery of islets and in contact with both endocrine and exocrine cells, or 3) in the vicinity of the islet within the estimated radius length of the closest islet. The areas of all islets in the VIP-stained sections were measured using ArcturusXT (Thermo Fisher Scientific, Waltham, USA) and Nikon Nis-Elements software. A total of 1810 islets were examined. On average 133 islets (SD = 71), 105 islets (SD = 38) and 28 islets (SD = 14) were counted in non-diabetic, recent onset type 1 diabetic and long-standing type 1 diabetic individuals, respectively. The area of the tissue analyzed was measured using ArcturusXT software. In each group investigated, the mean area of the analyzed sections was > 30 mm^2^.

### Statistical analysis

GraphPad Prism software (version 6.0d) was used for statistical analysis. The non-parametric Kruskal-Wallis test was used to compare the density of axons between different patient-groups. P < 0.05 was considered statistically significant.

## Results

### Parasympathetic axon density is decreased in the recent onset type 1 diabetic pancreas

**Fig 2:**
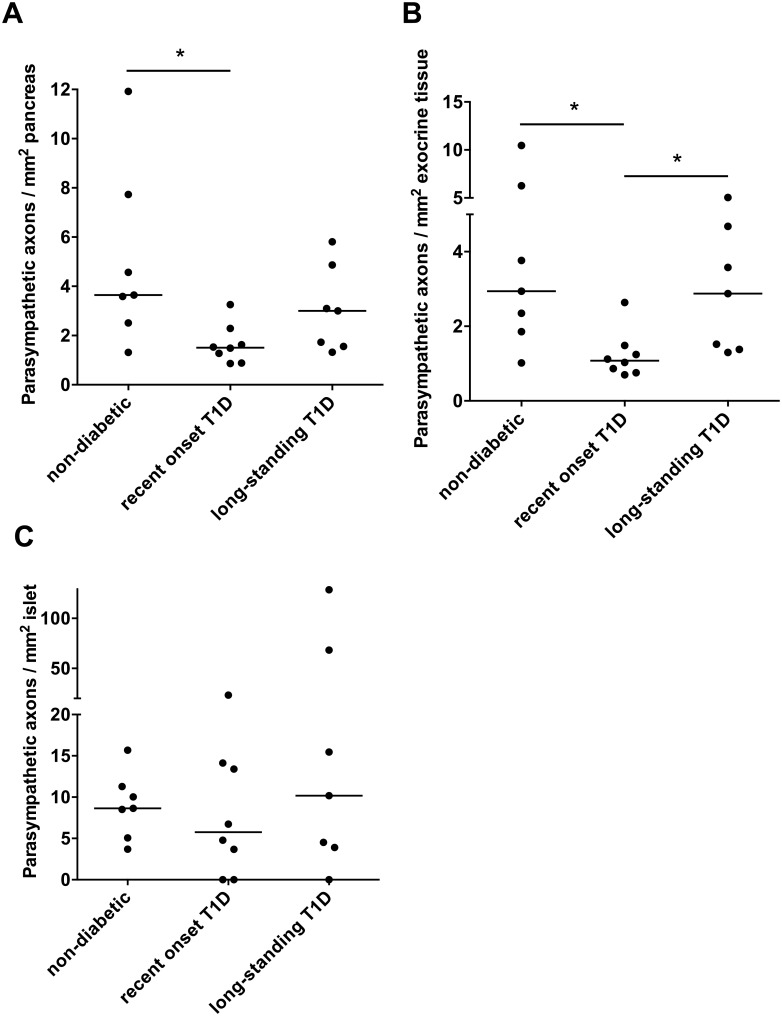
The density of parasympathetic axons in whole pancreas as well as in the exocrine and endocrine compartment. The density of parasympathetic axons in whole pancreatic tissue (A), exocrine pancreatic tissue (B), and within islets or in the periphery of an islet (C) of non-diabetic, recent onset type 1 diabetic, and long-standing type 1 diabetic pancreata. The bar in each group illustrates the median value. *, p<0.05.

VIP staining was intense within nerve trunks, demonstrating that parasympathetic axons were successfully labeled (Fig 1A and S1A and S1B Fig). Parasympathetic axons were also found in exocrine tissue (Fig 1B) and in association to islets (Fig 1C and S1C Fig) as well as in connective tissue, and around blood vessels and ducts. The density of parasympathetic axons was lower in pancreata from individuals with recent onset type 1 diabetes than from non-diabetic control individuals (Fig 2A; p = 0.023). Pancreata from individuals with long-standing type 1 diabetes had an intermediate density of parasympathetic axons that was not significantly different from any of the two other groups (Fig 2A).

### Parasympathetic axon density is reduced in the exocrine pancreas of individuals with recent onset type 1 diabetes

In the exocrine tissue of individuals with recent onset type 1 diabetes, the median density of parasympathetic axons was 1.08 (range 0.70–2.63) axons/mm^2^. The density was significantly lower than in the exocrine tissue of non-diabetic controls, which had 2.94 (range 1.02–10.47) axons/mm^2^ (p = 0.020), and donors with long-standing type 1 diabetes, which had 2.87 (range 1.30–5.07) axons/mm^2^ (p = 0.046; Fig 2B).

### Type 1 diabetes does not affect density of islet-associated parasympathetic axons

The location of parasympathetic axons in relation to islets was investigated. The islet-associated parasympathetic axons per islet (axons within or at the periphery of the islet) were not significantly different between the groups. The median amount of axons/islet was 0.05 (range 0.02–0.18) in the non-diabetic pancreata, 0.04 (range 0–0.15) in the recent onset type 1 diabetic pancreata, and 0.05 (range 0–0.74) in the long-standing type 1 diabetic pancreata. Similarly, there were no significant differences between the groups when investigating axon density in islets (Fig 2C). Furthermore, no significant difference could be discovered when the density of parasympathetic axons, or the parasympathetic axons per islet, were examined separately within, in the periphery, or in the close vicinity of islets.

## Discussion

A reduced density of parasympathetic axons in the exocrine pancreas of recent onset type 1 diabetes individuals was evident, whereas the density did not differ between long-standing type 1 diabetic and non-diabetic pancreata. Whether type 1 diabetes causes a reduced density of parasympathetic axons, or whether individuals with type 1 diabetes have a lower density of axons before onset of disease is at present unknown.

There are several factors that could induce axon degeneration e.g. viral, bacterial inflammatory or mechanical stimuli [21]. Hyperglycemia also causes neuropathy [22] but it appears unlikely to cause the reduced parasympathetic axon density of the pancreas in the group of recent onset type 1 diabetes individuals. On the other hand, long-standing type 1 diabetes patients are expected to have a higher risk of polyneuropathy due to hyperglycemia for an extended period of time but, in our study, no reduction of parasympathetic axons was found in the pancreas of this group. Unfortunately the medical records of the donors were not available (in order to protect the integrity of the deceased person), and therefore we could not determine whether these donors had any previous neurological complications.

As the lower density of parasympathetic axons was not sustained in long-standing type 1 diabetes, there is a possibility that the event that triggers type 1 diabetes is also harmful for parasympathetic axons and when this event subsides the axons reinnervate the pancreas. An alternative explanation to the higher density of axons in long-standing type 1 diabetes pancreata is a reduced pancreas volume but unaltered amount of axons that would consequently result in an elevated density of axons. The pancreas volume has been reported to be reduced in type 1 diabetes by several studies [23–26] but larger studies and longitudinal imaging studies are required to establish whether the pancreas is further reduced after diagnosis [27]. On the basis of the current knowledge it is therefore difficult to determine whether the amount of parasympathetic axons is elevated or unaltered in long-standing type 1 diabetes pancreata.

In animal models, parasympathetic axons contribute to the secretory function of the exocrine pancreas [28, 29] and in humans affected by type 1 diabetes, pancreas exocrine insufficiency is common [27]. It is conceivable that the reduced amount of parasympathetic axons leads to an impaired exocrine secretory function in type 1 diabetic patients. However, due to the limited knowledge of the normal function of parasympathetic innervation of the exocrine tissue [29], the exact impact of reduced parasympathetic axons in type 1 diabetes requires further investigations.

Previous studies report VIP immunoreactive axons to be located within and in the proximity to pancreatic islets, and also to exocrine structures of the pancreas [12–15]. By investigating more than 1800 islets, we found that only a small fraction of islets contained VIP immunoreactive axons. This suggests that the islets are sparsely innervated by parasympathetic axons. In agreement with our findings, Rodriguez-Diaz et al. suggested that parasympathetic axons, identified by staining for vAChT and ChAT, are rarely seen in the islets [9]. The exact innervation targets and the functional capacity of the axons have not been examined in this study. However, the density of parasympathetic axons within islets did not differ between pancreata from type 1 diabetic and non-diabetic individuals arguing for that parasympathetic innervation of the islets is not affected in type 1 diabetes.

The examination of the parasympathetic nervous branch within the pancreas was performed using immunofluorescence and morphometry. As thin sections were examined, only axons within a certain angle-span will have been included in the analysis, e.g. transected axons have been excluded whereas horizontal axons have been included. The risk for excluding axons was, however, minimized by manually investigating all focal planes of the sections. To exclude false positive staining, an axon was defined as at least three aligned immunoreactive spots. Also, the background was low in the examined tissues (as is illustrated in Fig 1 and S1 Fig), minimizing the risk of counting artifact immunoreactive spots as positive. The main aim of this study was to compare the axon density between type 1 diabetic and matched control pancreata. As this study was performed blinded, a relative comparison of the pancreatic parasympathetic axon density between controls and type 1 diabetic individuals was possible.

The access to human pancreatic tissue from individuals with recent onset type 1 diabetes is limited. This inevitably reduces the statistical power of the study and it is therefore possible that there are differences between the groups that could not be discovered. Nevertheless, the DiViD study includes pancreatic tissue from the largest cohort of well-characterized individuals with recent onset type 1 diabetes available so far. The DiViD samples were obtained from living patients, minimizing changes in the tissue due to brain death, ICU treatment and ischemia. The remaining samples were from brain dead organ donors, and were optimally preserved as for transplantation purposes, but the process of brain death and longer ischemia time may have introduced differences in parasympathetic axon density not related to diabetes. However, there was no tendency to a correlation between cold ischemic time and parasympathetic axon density and the reduced parasympathetic axon density was evident also in the two brain dead organ donors with recent onset type 1 diabetes.

In summary, this study demonstrates a reduction in density of parasympathetic axons in the exocrine pancreas examined shortly after onset of type 1 diabetes. Parasympathetic axons were found in islets to a similar extent in both non-diabetic and type 1 diabetic individuals. Further studies are required to assess the physiological role of the reduced density of parasympathetic axons in the exocrine pancreas of patients recently diagnosed with type 1 diabetes.

## Supporting information

S1 Fig Images of a nerve bundle within pancreatic tissue excited with 488nm laser. A: Autofluoresence above the emission spectrum for Cy2. B: Emission over the entire spectrum. Emission within the spectrum for Cy2, that was conjugated to the VIP antibody, is seen in green. C: The same nerve bundle in lower magnification. Four islets can be identified based on their autofluoresence and morphometry on the right side of the nerve bundle. The scale bars correspond to 50 μm.
(TIF)
