# Supplementary figures and images for: The density of parasympathetic axons is reduced in the exocrine pancreas of individuals recently diagnosed with type 1 diabetes
Source: PLoS One. 2017 Jun 19;12(6):e0179911. doi: 10.1371/journal.pone.0179911 (PMC5476281; doi:10.1371/journal.pone.0179911)

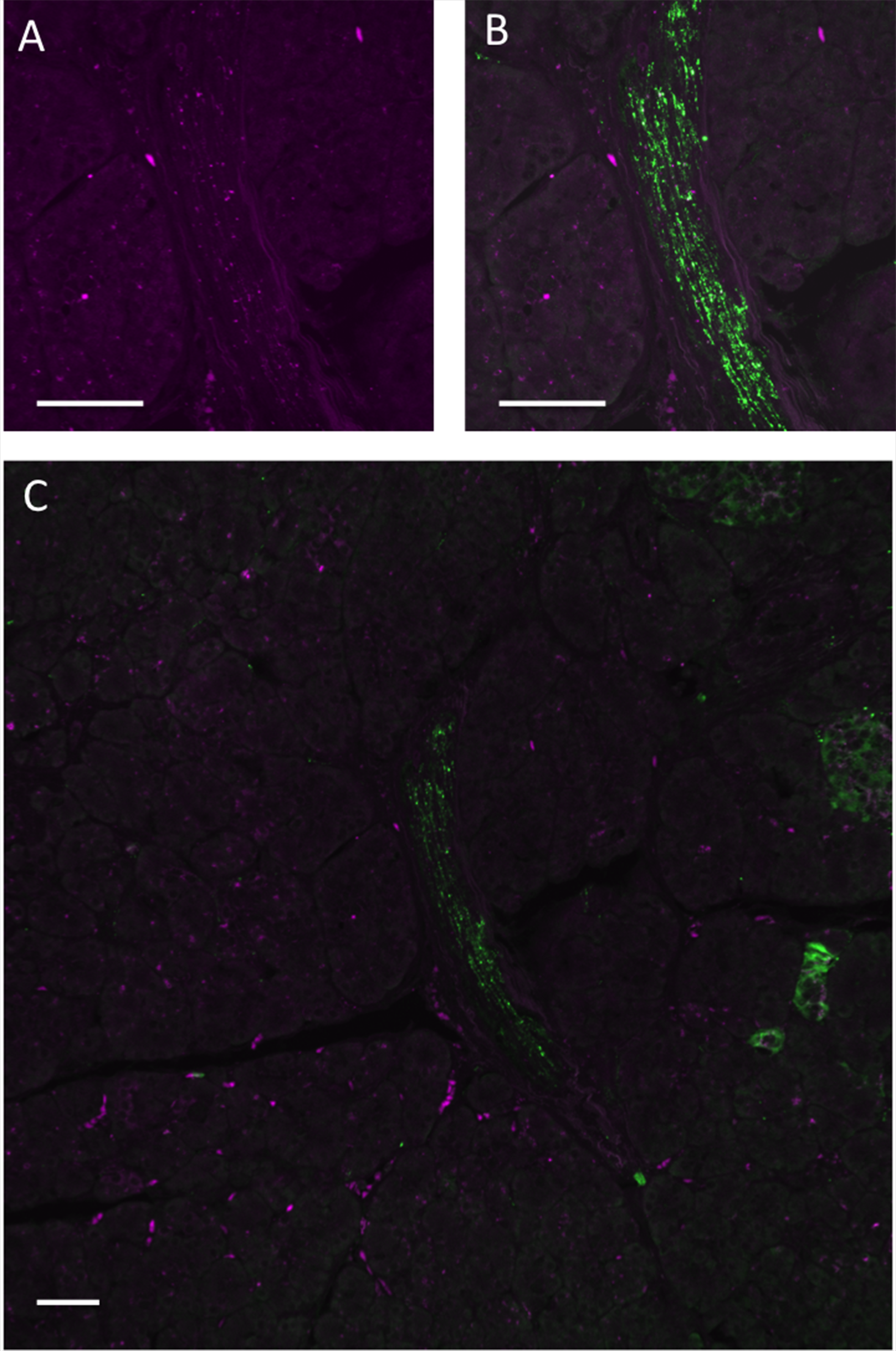

Supplement: S1 Fig — A: Autofluoresence above the emission spectrum for Cy2. B: Emission over the entire spectrum. Emission within the spectrum for Cy2, that was conjugated to the VIP antibody, is seen in green. C: The same nerve bundle in lower magnification. Four islets can be identified based on their autofluoresence and morphometry on the right side of the nerve bundle. The scale bars correspond to 50 μm. (TIF) [file pone.0179911.s001.tif]
